# Novel interaction of properdin and coagulation factor XI: Crosstalk between complement and coagulation

**DOI:** 10.1002/rth2.12715

**Published:** 2022-05-24

**Authors:** Samantha L. Heal, Lewis J. Hardy, Clare L. Wilson, Majid Ali, Robert A. S. Ariëns, Richard Foster, Helen Philippou

**Affiliations:** ^1^ 4468 Discovery and Translational Science Department Leeds Institute of Cardiovascular and Metabolic Medicine University of Leeds Leeds UK; ^2^ 4468 School of Chemistry University of Leeds Leeds UK

**Keywords:** coagulation factor, complement, complement system, enzyme kinetics, factor XI (FXI), polyanion, properdin (FP), substrate specificity, surface plasmon resonance (SPR)

## Abstract

**Background:**

Evidence of crosstalk between the complement and coagulation cascades exists, and dysregulation of either pathway can lead to serious thromboinflammatory events. Both the intrinsic pathway of coagulation and the alternative pathway of complement interact with anionic surfaces, such as glycosaminoglycans. Hitherto, there is no evidence for a direct interaction of properdin (factor P [FP]), the only known positive regulator of complement, with coagulation factor XI (FXI) or activated FXI (FXIa).

**Objectives:**

The aim was to investigate crosstalk between FP and the intrinsic pathway and the potential downstream consequences.

**Methods:**

Chromogenic assays were established to characterize autoactivation of FXI in the presence of dextran sulfate (DXS), enzyme kinetics of FXIa, and the downstream effects of FP on intrinsic pathway activity. Substrate specificity changes were investigated using SDS‐PAGE and liquid chromatography–mass spectrometry (LC‐MS). Surface plasmon resonance (SPR) was used to determine direct binding between FP and FXIa.

**Results/Conclusions:**

We identified a novel interaction of FP with FXIa resulting in functional consequences. FP reduces activity of autoactivated FXIa toward S‐2288. FXIa can cleave FP in the presence of DXS, demonstrated using SDS‐PAGE, and confirmed by LC‐MS. FXIa can cleave factor IX (FIX) and FP in the presence of DXS, determined by SDS‐PAGE. DXS alone modulates FXIa activity, and this effect is further modulated by FP. We demonstrate that FXI and FXIa bind to FP with high affinity. Furthermore, FX activation downstream of FXIa cleavage of FIX is modulated by FP. These findings suggest a novel intercommunication between complement and coagulation pathways.

**Essentials**

- Dysregulation of complement and coagulation go hand in hand and contribute to thromboinflammation.
- We explored crosstalk of complement factor properdin (FP) and intrinsic coagulation factor XI (FXI).
- FP is cleaved by activated FXI (FXIa), binds to FXIa with high affinity, and modulates FXIa activity.
- FP may modulate surface‐driven FXIa activity with downstream functional consequences.

## 1 INTRODUCTION

The complement and coagulation pathways play a central role in thromboinflammation. Both systems are descended from shared ancestry,^1^ and although the interactions between the two are yet to be completely defined, the two systems should not be considered as separate entities.^2^

Complement is a tightly regulated protease cascade and is a key player in host defense against microbial infection. This cascade can be activated by three pathways: the classical, lectin, and alternative pathways. These three pathways result in a common terminal pathway, culminating in membrane attack complex (MAC) formation.^3^

The classical and lectin pathways are inducible through conformational changes in the primary proteases through antibody‐antigen complexes^4^ or when in contact with polysaccharides at microbial surfaces.^5^

The alternative pathway is different from the inducible classical and lectin pathways, constantly undergoing a process known as “tick‐over,” the spontaneous hydrolysis of the thioester bond of C3 creating C3(H_2_O), an analogue of C3b.^6^ C3(H_2_O) will either bind to pathogen surfaces to recruit further complement components or will remain soluble and be quickly degraded. Properdin (factor P [FP]) and factor B (FB) are recruited, forming a complex with surface bound C3(H_2_O), with FB binding in a Mg^2+^‐dependent manner. FB is then activated by factor D, forming the initial C3 convertase. FP is a cofactor for the C3 convertase complex, increasing the half‐life 10‐fold,^7^ and is the only known positive regulator of complement, necessary for alternative pathway activation, and initiates a positive feedback loop, amplifying the terminal pathway and MAC generation culminating in lysis of pathogenic cells.^8^

FP is a highly positively charged 53‐kDa monomer made of seven thrombospondin type‐1 repeats. These monomers associate head to tail to create dimers, trimers, and tetramers in serum.^9^ FP circulates in the plasma at a concentration of 4 to 25 µg/mL and is constitutively released from a number of cells^10^ including monocytes,^11^ dendritic cells,^12^, ^13^ endothelial cells,^10^ mast cells,^14^ and adipocytes.^15^, ^16^ FP is also released from stimulated granulocytes, including neutrophils, in the local microenvironment, promoting complement activation; stimuli include tumor necrosis factor‐α and C5a.^17^, ^18^ FP deficiency often displays a phenotype of recurring meningococcal infections, with higher mortality rates when compared to healthy individuals.^19^

The intrinsic pathway of coagulation is initiated through contact activation. Prekallikrein (PK) and factor XII (FXII) can undergo a reciprocal activation process enhanced by the presence of artificial and physiological negatively charges surfaces^20^ to generate kallikrein (PKa) and activated FXII (FXIIa). FXIIa generated from contact activation initiates the intrinsic pathway of coagulation through the cleavage of FXI to form FXIa. FXIa,^21^ along with PKa,^22^, ^23^, ^24^, ^25^ can then cleave factor IX (FIX), subsequently leading to factor X (FX) activation, thrombin generation, and fibrin clot formation. It has been demonstrated that in the presence of surfaces such as sulfatides and glycosaminoglycans (GAGs), FXI also can autoactivate and induce coagulation.^26^

FXI is a zymogen that circulates at a concentration of 30 nM in the blood, most often in association with its cofactor, high‐molecular‐weight kininogen (HK).^27^ FXI is composed of two identical 80‐kDa monomers linked by a disulfide bond. Each monomer contains four apple domains with similar structural properties to PK, which also circulates in complex with HK.^28^ Deficiency of FXI results in a mild to moderate bleeding disorder known as hemophilia C. FXI can be activated by FXIIa^29^, ^30^ and thrombin,^31^, ^32^ and FXIa has been shown to have numerous natural substrates including FIX, factor V, FX, prochemerin, and complement regulatory protein factor H (FH).^33^, ^34^, ^35^

Proteases from both the complement and contact activation systems have similar structural functions and characteristics, and it has been demonstrated that the classical pathway of the complement system can be initiated by FXIIa.^36^ Also, the common pathway of complement can be initiated through the cleavage of C3 by PKa.^37^ There is evidence that the lectin pathway of complement may interact with the kallikrein‐kinin system through mannose‐binding lectin‐associated serine protease‐1 cleavage of HK leading to bradykinin release.^38^ The primary inhibitor of the complement classical pathway, C1 esterase inhibitor (C1‐INH) can also inhibit the intrinsic pathway of coagulation^39^ through inhibition of FXIIa, FXIa, and PKa,^40^ demonstrating how both systems are intricately related and have functional similarities.

Polyanions including GAGs, polyphosphates, and phospholipids are key regulators of both complement and coagulation.^41^ They can induce contact activation of coagulation resulting in intrinsic activation, which can lead to initiation of the alternative pathway of complement. Some can also modulate inhibition of coagulation and complement via potentiating serpin activity.^42^ Phospholipids can mediate both complement and coagulation, and FXII can bind to apoptotic cells via phospholipid interactions,^43^ while the alternative pathway of complement can also activate upon binding to phospholipid surfaces.^44^

In this study, we have demonstrated for the first time that FXIa and FP have a direct interaction leading to functional consequences, revealing a novel intercommunication of the coagulation and complement pathways. These findings may have important implications in pathophysiological mechanisms involving intrinsic pathway and complement activation in thromboinflammatory conditions such as disseminated intravascular coagulation.

## 2 MATERIALS AND METHODS

### 2.1 Materials

Human FP was sourced from Complement Technologies Inc. (Tyler, TX, USA) Human FXI, FIX, and FX zymogens and active enzyme FXIa (preactivated by FXIIa) were obtained from Haematologic Technologies Inc. (Essex Junction, VT, USA). Human single‐chain HK was sourced from Enzyme Research Laboratories Ltd. (Llansamlet, UK). Dextran sulfate sodium salt from *Leuconostoc* sp. (500 kDa) was sourced from Sigma‐Aldrich Corporation (Merck Group, St. Louis, MO, USA). Amine coupling reagents were obtained from GE Healthcare (Chicago, IL, USA). A Slide‐a‐Lyzer MINI dialysis device (MWCO 7000 Da was obtained from Life Technologies (Thermo Fisher, Waltham, MA, USA). Chromogenic substrates S‐2288 and S‐2765 were obtained from Quadratech Diagnostics Ltd. (East Sussex, UK). Four percent to 12% Bis‐Tris Gels, 4× sample buffer, 10× LDS reducing buffer and 20× MES running buffer were all obtained from Thermo Fisher Life Technologies. Precision Plus Protein Dual Colour Standard was obtained from Bio‐Rad Laboratories (Hercules, CA, USA). InstantBlue Protein Stain was obtained from Expedeon Ltd. (Cambridge, UK). All buffer ingredients were sourced from Sigma‐Aldrich Corporation (Merck Group), unless otherwise stated. Phospholipids were obtained from Avanti Polar Lipids (Alabaster, AL, USA).

### 2.2 FXI chromogenic assay

All chromogenic assays were performed using half‐volume clear flat‐bottom polystyrene 96‐well plates (Fisher Scientific Ltd., Waltham, MA, USA) and 500 µM Chromogenix S‐2288 chromogenic substrate with 4‐(2‐hydroxyethyl)‐1‐piperazineethanesulfonic acid (HEPES) buffered saline pH 7.4 with 1% (w/v) PEG_8000_ (HBS [10 mM HEPES, 150 mM NaCl]‐P) unless otherwise stated. Cleavage of S‐2288 was determined by absorbance changes of the reaction mixture as p‐nitroaniline is released. All experiments were run in triplicate on one plate and were exported to Excel (Microsoft Corporation, Redmond, WA, USA). Readings were taken using a PowerWave HT Microplate Spectrophotometer (BioTek, Winooski, VT, USA) at 37°C at 405 nm every 12 seconds unless otherwise stated.

#### 2.2.1 FXI autoactivation

FP was titrated (5‐25 µg/mL) in reactions of 30 nM FXI with 0.6 µg/mL dextran sulfate (DXS). The generation of FXIa formed was subsequently determined by monitoring cleavage of S‐2288 every 12 seconds, over 120 minutes at 37°C. A similar assay was performed, replacing the titration of FP with a titration of protamine sulfate (0.78–50 µg/mL; Figure S1).

#### 2.2.2 Michaelis‐Menten kinetics

Three nanoMolar of FXIa was incubated with and without 0.6 µg/mL DXS, in the presence and absence of 25 µg/mL FP using a twofold serial dilution of S‐2288 (0.05‐3.0 mM). FXIa catalytic activity was determined by monitoring cleavage of S‐2288.

### 2.3 Substrate specificity assays

Substrate specificity was analyzed using reducing SDS‐PAGE. Incubations were performed and all samples were diluted twofold into a running buffer containing reducing agent, LDS sample buffer, and HBS. Samples were separated by reducing SDS‐PAGE at 100 V for 52 minutes. The gel was stained with InstantBlue protein stain overnight at 21°C with gentle shaking, and washed three times with water with gentle shaking for 5 minutes. Gels were imaged using Syngene G:BOX Chemi and GeneSys software (Syngene, Bengaluru, India).

#### 2.3.1 FXI in the presence of DXS and FP

Reactions of 100 µg/mL FXIa and 200 µg/mL FP in the presence of 12.5 µg/mL DXS were incubated for 120 minutes in HBS and were diluted into the running buffer. Bands of interest were analyzed by liquid chromatography–mass spectrometry (LC‐MS) (Appendix S1) by the Biomolecular Mass Spectrometry Facility at the University of Leeds.

#### 2.3.2 FXIa substrate specificity

Reactions of 100 µg/mL FXIa, 100 µg/mL FIX, and 200 µg/mL FP in the presence of 12.5 µg/mL DXS were incubated, and samples were taken over a course of 60 minutes and were diluted into the running buffer.

### 2.4 Binding of FXI and FP using surface plasmon resonance

SPR was performed using the Pall/ForteBio Pioneer biosensor platform (Molecular Devices, LLC, San Jose, CA, USA) (Appendix S1). FP was immobilized to the sensor surface to around 1 × R_MAX_ 79.55 RU using the amine coupling method as previously described.^45^

After priming three times, 50 nM of FXI or FXIa was injected over the sensor surface for the first experiment using a OneStep 100% loop inject, using Taylor dispersion to create a concentration gradient through the capillary tube before entering the flow cell (FC), at a flow rate of 30 µL/min with a dissociation time of 300 seconds. The sensor surface was regenerated using 5 µL of 1 M NaCl, 3 mM NaOH injected at 60 µL/min, with a dissociation time of 30 seconds.

To determine the binding kinetics, a OneStep assay was performed. Response curves from FC2 were subtracted from FC1 and FC3. Buffer blanks were averaged and subtracted from the binding curves. The assay was run in triplicate, and the standard error of the mean was calculated using Prism 8 (GraphPad Software, San Diego, CA).

### 2.5 FXIa downstream activity chromogenic assay

#### 2.5.1 Phospholipid vesicle preparation

Phospholipids were prepared as previously described.^46^ Micelles were formed from phospholipids supplied in chloroform, using 20% (v/v) di‐oleic phosphatidylethanolamine, 20% (v/v) di‐oleic phosphatidylserine, and 60% (v/v) di‐oleic phosphatidylcholine dehydrated and resuspended in HBS.

Reactions of 125 pM FXIa, 100 nM FIX, 300 nM FX, 125 pM HK, 100 µM phospholipids, and 25 µg/mL FP were analyzed using a chromogenic assay to determine FX activation downstream of FXIa activation of FIX. FX activation was measured by monitoring cleavage of 700 µM chromogenic substrate S‐2765 at 37°C at 405 nm every 12 seconds for 360 minutes. Maximum rates of reactions were calculated from the first derivatives of each assay condition.

### 2.6 Data analysis and statistics

All data figures were created using GraphPad Prism 8 unless otherwise stated.

#### 2.6.1 Statistical analysis

All analysis was performed in GraphPad Prism 8 unless otherwise stated. Statistical differences were analyzed by one‐way analysis of variance (anova), and tests are highlighted in figure legends where appropriate.

#### 2.6.2 Surface plasmon resonance

K_D_ was calculated using Qdat data analysis software package (Pall FortéBio, Menlo Park, CA, USA), specific for Molecular Devices Pioneer data sets.

## 3 RESULTS

### 3.1 Amidolytic activity of FXI autoactivated by DXS is modulated in the presence of FP

**FIGURE 1:**
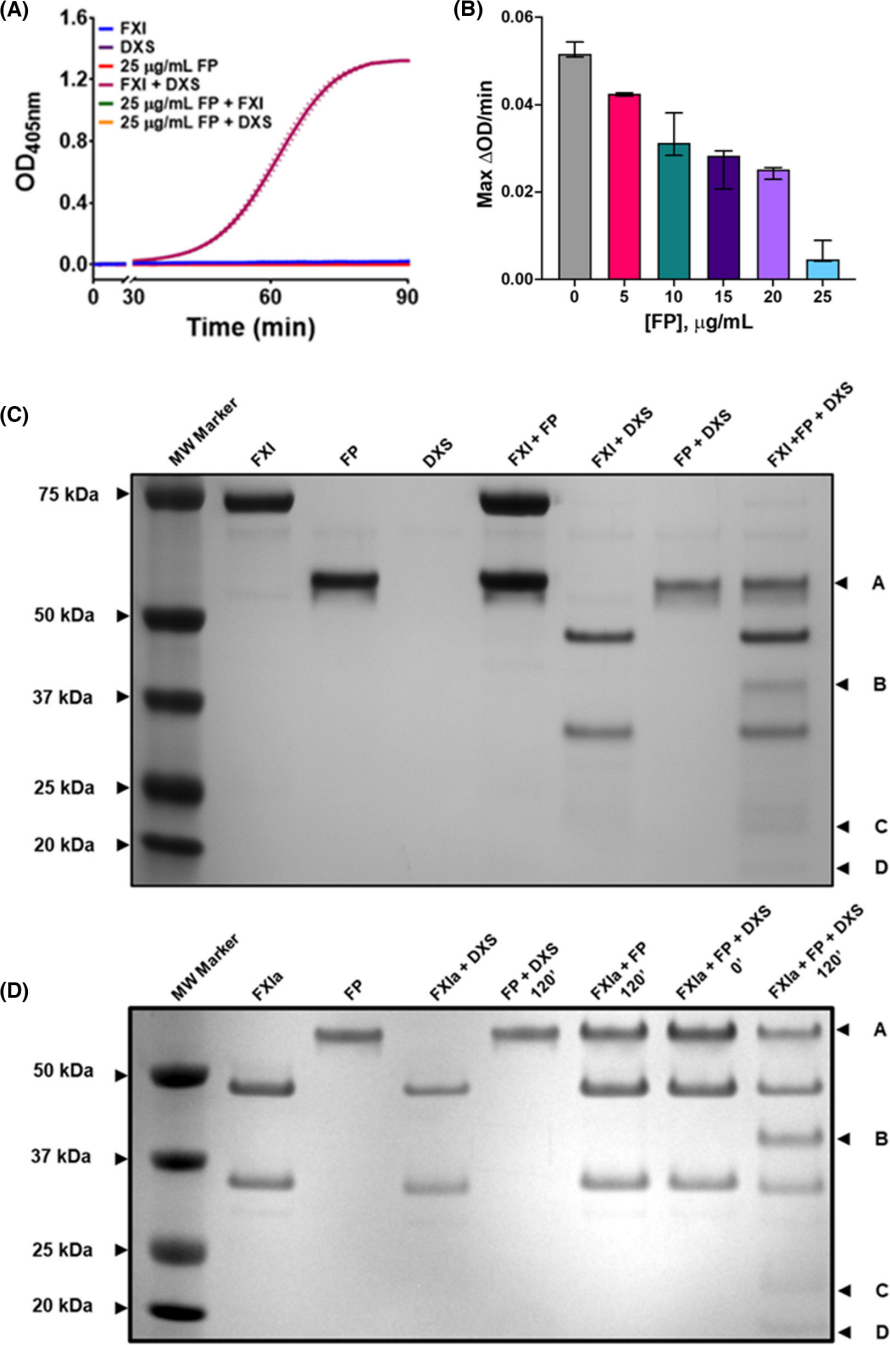
FP acts as a substrate for FXIa in the presence of DXS. A chromogenic assay was employed to determine the effect of FP on the autoactivation of 30 nM FXI by 0.6 μg/mL DXS. FXI autoactivation was measured by cleavage of 500 μM chromogenic substrate S‐2288 over 90 minutes. (A) Controls: FXI only (blue), DXS only (purple), FP only (red), FXI + DXS (maroon), FP + FXI (no DXS, green), FP + DXS (no FXI, orange). (B) FXI autoactivation by DXS in the presence of a titration of physiological concentrations of FP (5–25 μg/mL). The maximum rate of reaction calculated using the first derivative. Optical density was read at 405 nm, at 12 second intervals at 37°C. Data are expressed as median ±interquartile range, constructed of one experiment run in triplicate. (C) SDS‐PAGE reveals a substrate specificity change of FXIa toward FP. (D) Purified FXIa can cleave FP in the presence of DXS. Bands A, B, C, and D were analyzed by mass spectrometry and were revealed to be FP (Table S1). Abbreviations: DXS, dextran sulfate; FP, factor P; FXI, factor XI; FXIa, activated factor XI

This study was initiated by investigating the effect of FP on FXI autoactivation by the synthetic GAG DXS. In a purified system, physiological concentrations of FP and FXI were incubated in the presence and absence of DXS for 90 minutes. The presence of DXS rapidly induced amidolytic activity of FXI toward S‐2288 via autoactivation to FXIa (Figure 1A). The addition of FP (5–25 µg/mL) to the autoactivation reaction drastically reduced cleavage of S‐2288 in a dose‐dependent manner (Figure 1B). To determine if this effect was due to the cationicity of FP, the same experiment was repeated using a titration of the cationic protein protamine sulfate, a clinical reagent applied for the reversal of heparin anticoagulation.^47^ It was observed that protamine sulfate reduced the cleavage of chromogenic substrate by FXI autoactivation in the presence of DXS, though this was not dose dependent with the employed concentrations (Figure S1). To determine if the decreased cleavage of the chromogenic substrate was due to a change in substrate specificity, reactions of FXI, DXS, and FP were incubated for 120 minutes and were analyzed using reducing SDS‐PAGE. Novel cleavage bands were revealed and were analyzed by LC‐MS (Table S1). The cleavage products were found to be FP, suggesting a change in substrate specificity of FXIa away from the chromogenic substrate and toward FP. Similar reactions were performed and analyzed using SDS‐PAGE; however, FXI was replaced with its activated form, purified FXIa (preactivated by FXIIa) (Figure 1D). The cleavage products of FP were still visible, suggesting that FP can be cleaved by FXIa autoactivated by DXS, or by FXIa activated by FXIIa. These data suggest a surface‐dependent interaction between FXI and FP.

### 3.2 FXIa is still able to cleave its natural substrate, FIX, in the presence of FP and DXS

**FIGURE 2:**
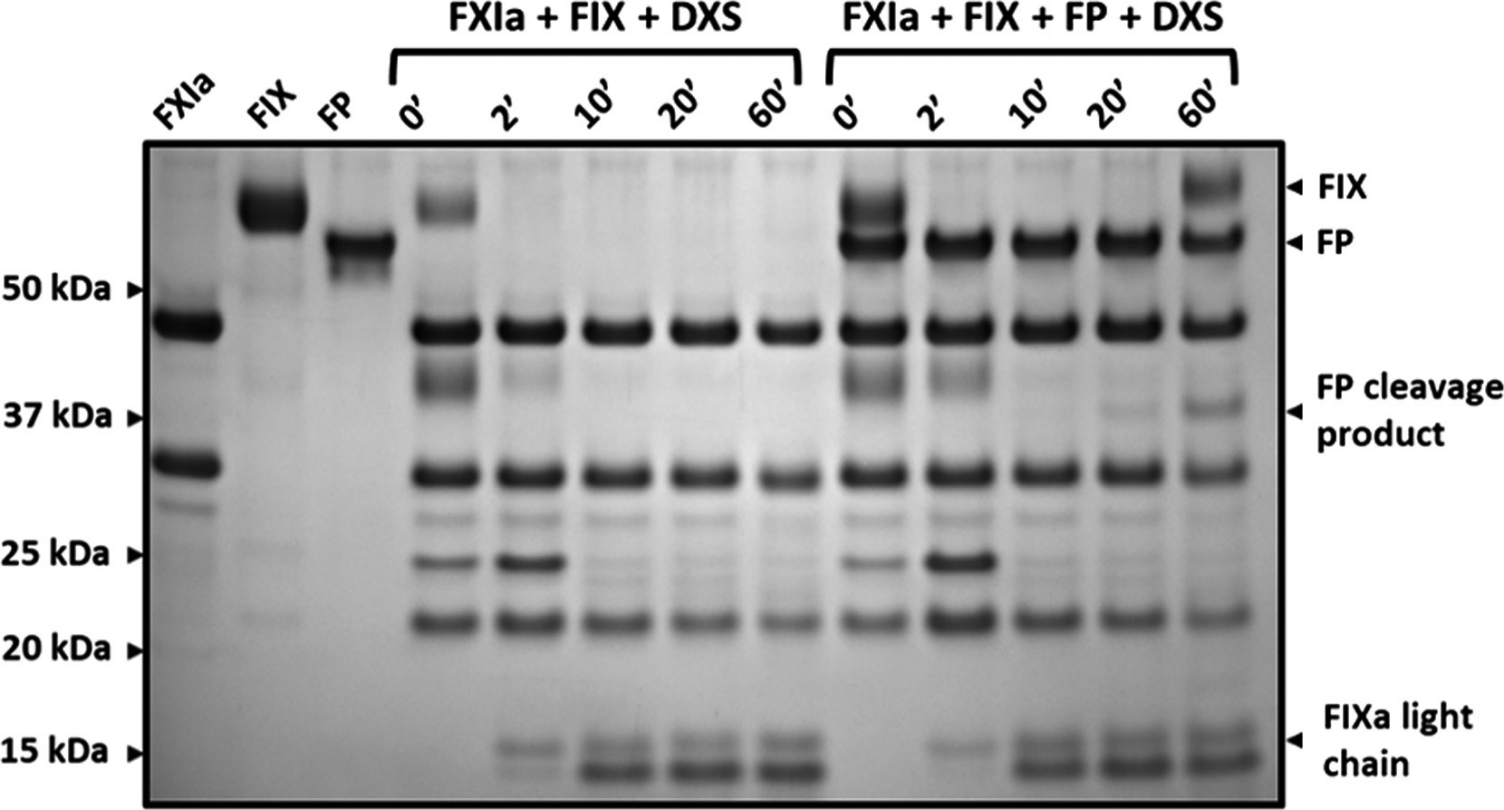
FXIa can cleave its physiological substrate, FIX, and FP in the presence of DXS. Reactions of 100 μg/mL FIX, 100 μg/mL FXIa, 200 μg/mL FP, and 12.5 μg/mL DXS were incubated at 37°C for up to 60 minutes with samples taken intermittently after briefly mixing with a vortex. Reducing SDS‐PAGE was performed to analyze FXIa substrate specificity. Activation of FIX was determined by the appearance of the FIXa light chain, cleavage of FP was determined by the appearance of the heaviest cleavage product at around 40 kDa. Abbreviations: DXS, dextran sulfate; FIX, factor IX; FIXa, activated factor IX; FP, factor P; FXI, factor XI; FXIa, activated factor XI

It was important to investigate the effect of FP on cleavage of FIX by FXIa in the presence of DXS to elucidate whether the changes in substrate specificity may implicate the activation of coagulation via the intrinsic pathway. Reactions of FXIa, FIX, FP, and DXS were incubated as a time course, for up to 60 minutes. The samples were analyzed using reducing SDS‐PAGE. It was indicated that FXIa was able to cleave both FIX and FP in the presence of DXS (Figure 2).

### 3.3 DXS modulates the kinetics of FXIa, but this is further modified in the presence of FP

Subsequently, we explored FXIa activity in the presence and absence of DXS and FP (Table 1). The data were analyzed using the *k*_cat_ model in GraphPad Prism. DXS alone reduced the turnover rate of S‐2288, demonstrated by the decrease in maximum velocity (V_max_) of FXIa toward the substrate from 6.3 × 10^−7^ to 1.6 × 10^−7^ (M.s^−1^). This reduced turnover rate observed at concentrations above the *K*_m_ is due to a significant reduction in *k*_cat_ from 209.5 to 51.8 s^−1^, which was also compensated for by a significant decrease in *K*_m_ 4.9×10^−4^ to 3.3×10^−5^ M. The decrease in both *k*_cat_ and *K*_m_ leads to an overall increase in *k*_cat_/*K*_m_. The addition of FP alone did not modulate FXIa activity; however, it reversed the reduction of *k*_cat_ by DXS from 51.76 to 88.4 s^−1^ and partially reversed the decrease in *K*_m_ from 2.1×10^−4^ M to 3.3×10^−5^. These data suggest that FP is most likely interfering with the interaction between FXIa and the polyanionic surface presented by DXS; however, the partial reversal of the reducing effect on *K*_m_ supports a potential substrate specificity change of FXIa.

**TABLE 1:** FP reduces the inhibitory effect of DXS on FXIa catalytic activity

	*k*_cat_ ± SEM (s^−1^)	*K*_m_ ± SEM (M)	V_max_ (M.s^−1^)	*k*_cat_/*K*_m_ (s^−1 ^M^−1^)
FXIa	209.5 ± 3.8	4.9 × 10^−4^ ± 2.6 × 10^−5^	6.3 × 10^−7^	4.24 × 10^5^
FXIa + FP	196.1 ± 5.6	4.3 × 10^−4^ ± 3.7 × 10^−5^	5.9 × 10^−7^	4.61 × 10^5^
FXIa + DXS	51.8 ± 4.3	3.3 × 10^−5^ ± 1.5 × 10^−5^	1.6 × 10^−7^	1.59 × 10^6^
FXIa + FP + DXS	88.4 ± 6.9	2.1 × 10^−4^ ± 5.9 × 10^−4^	2.7 × 10^−7^	4.14 × 10^5^

Note

Michaelis‐Menten kinetic analysis was performed to determine how FP affected FXIa catalytic activity in the presence and absence of DXS. Reactions of 25 µg/mL FP and 3 nM FXIa were incubated ±0.6 µg/mL DXS with a twofold titration of S‐2288 (0.05–3 mM). FXIa catalytic activity was measured by cleavage of S‐2288 over 3 h. Optical density was read at 405 nm, at 12‐s intervals at 37°C. Kinetic analysis was performed using the *k*_cat_ model on GraphPad Prism. Catalytic activity defined by amount of pNA released per second (M.s^−1^). Data represented as mean ± SEM constructed of one experiment run in triplicate.

Abbreviations: DXS, dextran sulfate; FP, factor P; FXIa, activated factor XI; *k*_cat_, first‐order rate constant; *K*_m_, Michaelis constant; pNA, p‐nitroaniline; SEM, standard error of the mean.

### 3.4 FXI and FXIa bind to FP with high affinity

**FIGURE 3:**
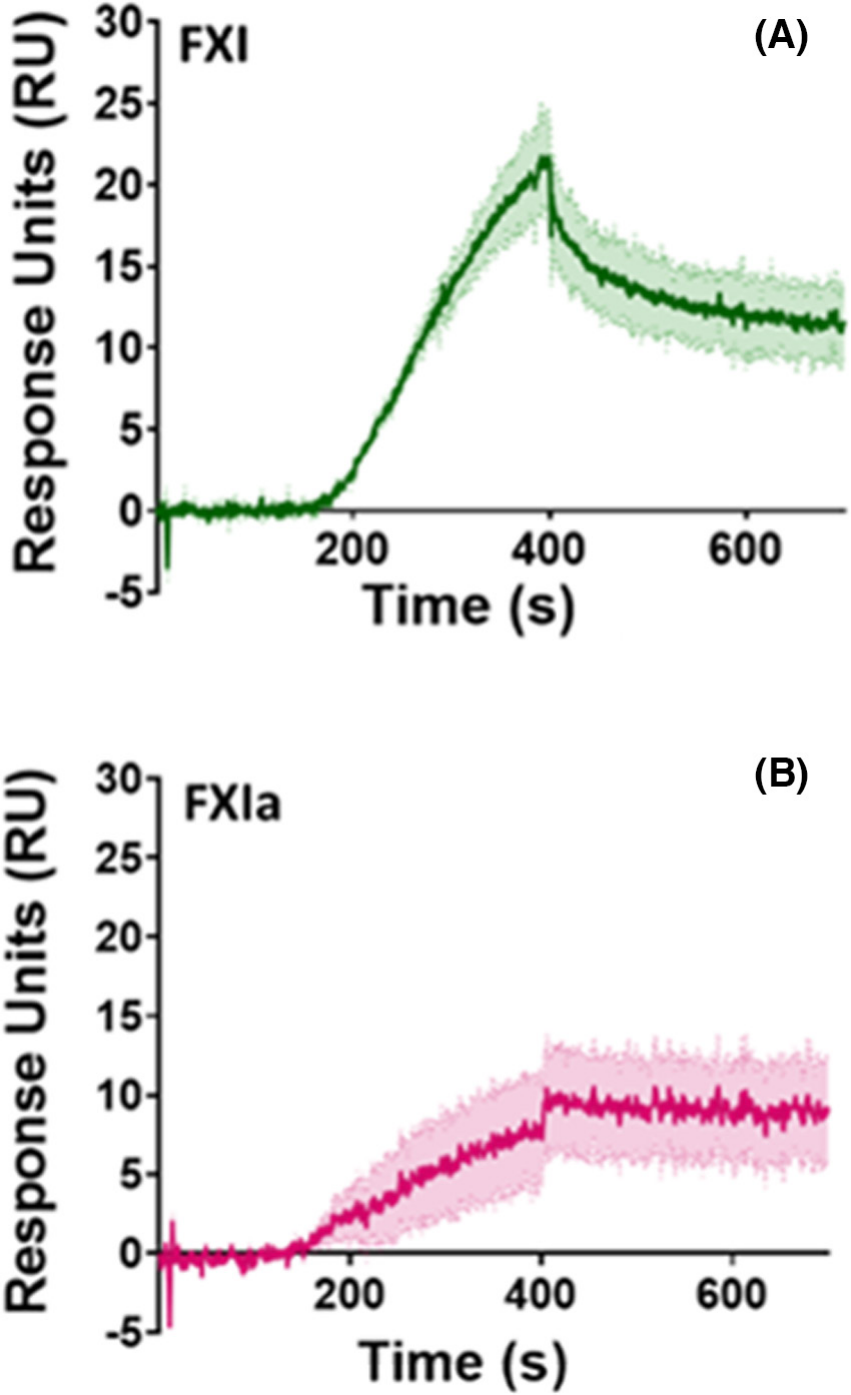
FXI and FXIa bind to FP with high affinity. Kinetic analyses were performed by SPR with human FP immobilized to the sensor surface to 79.55 RU. (A) 50 nM FXI was injected and bound to FP with a K_D_ of 16.1 nM. (B) 50 nM FXIa was injected and bound to FP with a K_D_ of 350 pM. Data are expressed as mean ± SEM constructed of one experiment run in triplicate. Abbreviations: FP, factor P; FXI, factor XI; FXIa, activated factor XI; K_D_, equilibrium dissociation constant; SPR, surface plasmon resonance

Binding studies were performed using SPR to determine if there was a direct interaction between FXIa and FP. FP was immobilized to the sensor surface and FXI and FXIa were titrated over the surface using the OneStep protocol, with a maximum concentration of 50 nM. We found that both FXI and FXIa bind to FP with a K_D_ (equilibrium dissociation constant) of 16.1 nM and 350 pM, respectively (Figure 3A, B). These data suggest that FP can bind to both activated and zymogen FXI but with a preference to the active enzyme due to a higher‐affinity interaction.

### 3.5 FP modulates FX generation by the intrinsic pathway in purified reactions

The purpose of this experiment was to measure FXIa activation of FIX; however, due to the low catalytic activity of FIXa toward chromogenic substrates, this was performed indirectly by observing FX activation by FIXa. The FX concentration (300 nM) is at the concentration of the *K*_m_ of FIXa toward FX, and the concentration of the chromogenic substrate S‐2765 (700 µM) is at the V_max_ of FXa; FX cleaving the substrate is not the rate‐limiting factor. The rate‐limiting factors of this assay would therefore be FXIa activation of FIX, or FIXa activation of FX.

**FIGURE 4:**
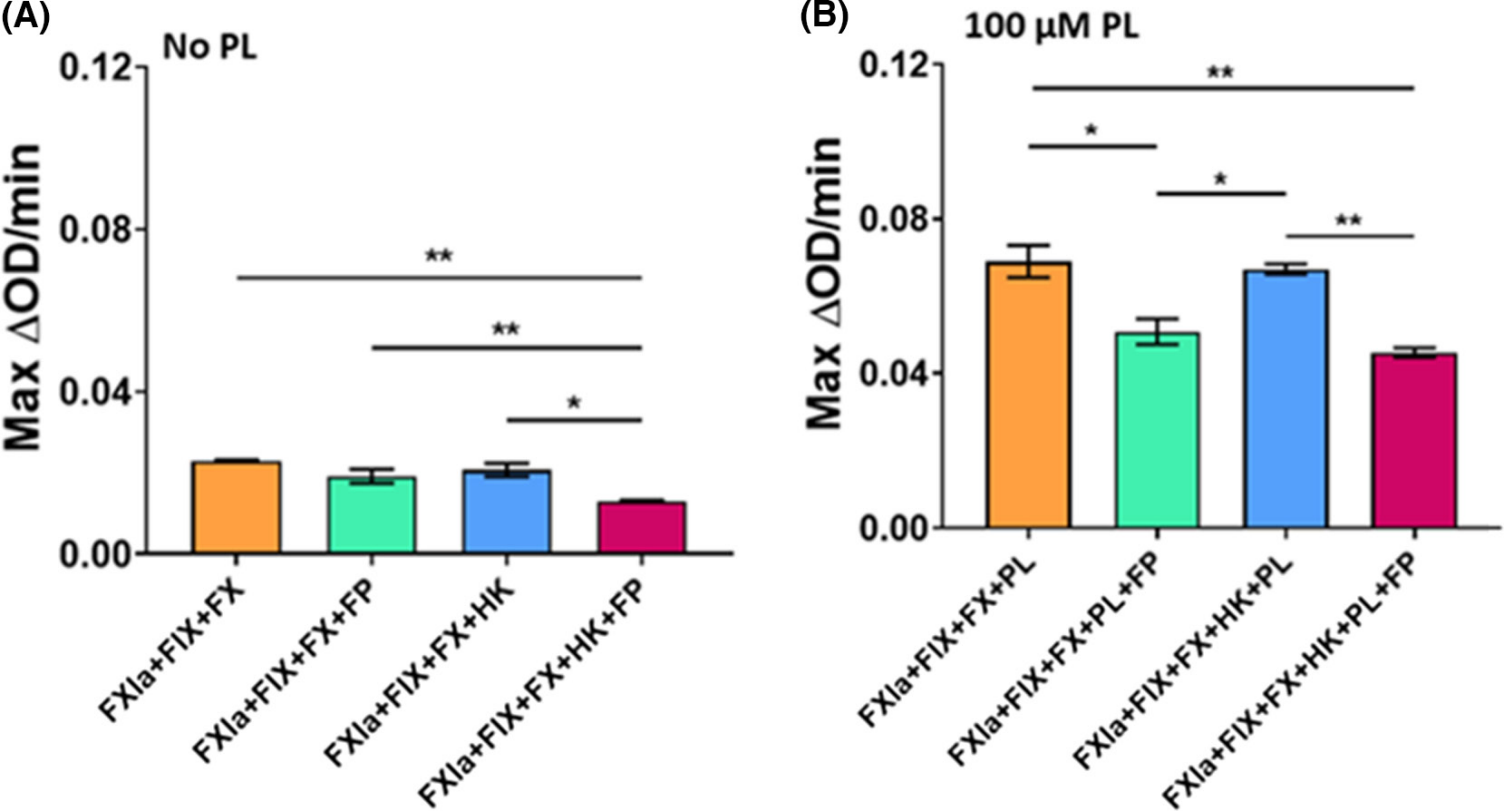
FP modulates downstream effects of FXIa by leading to reduced cleavage of chromogenic substrate S‐2765 by FXa. Reactions of 125 pM FXIa, 100 nM FIX, 300 nM FX, 125 pM HK, 100 μM PL, and 25 μg/mL FP were analyzed using a chromogenic assay to determine FX activation downstream of FXIa activation of FIX. FXa catalytic activity was measured by cleavage of 700 μM chromogenic substrate S‐2765 for 360 minutes. Optical density was read at 405 nm, at 12‐second intervals at 37°C. Maximum rates of reactions were calculated from the first derivatives of each assay condition. (A) Maximum rate of reaction calculated using the first derivative of the reactions in the absence of 100 μM PL. (B) Maximum rate of reaction calculated using the first derivative of the reactions performed in the presence of 100 μM PL. Data represented as mean ± standard error of the mean constructed of one experiment run in triplicate. Statistical differences were analyzed by one‐way analysis of variance, and differences between columns were detected by Tukey’s multiple comparisons test. *P ≤ .05, **P ≤ .01. Abbreviations: FIX, factor IX; FP, factor P; FX, factor X; FXa, activated factor X; FXIa, activated factor XI; HK, high‐molecular‐weight kininogen; PL, phospholipids

The effect of FP on the resulting activation of FX by FIXa was explored, in the presence and absence of HK and phospholipids. In the absence of phospholipids (Figure 4A), HK appeared to have little effect on the cleavage of S‐2765 in the absence of FP. However, in reactions including FP with HK, the cleavage of S‐2765 was significantly reduced when compared to all other assay conditions without phospholipids (*P *≤ .05). The addition of 100 µM of phospholipids enhances FXa generation in all conditions (Figure 4B); however, there is a similar trend where FP reduces the cleavage of the chromogenic substrate in the presence and absence of HK (*P *≤ .05).

## 4 DISCUSSION

Complement‐coagulation crosstalk has been a topic of research for over 80 years^48^; however, the mechanisms behind this are still not fully understood, and many thrombotic and inflammatory diseases involve both cascades. Moreover, it is important to understand how agents targeting coagulation may modulate inflammatory pathways and vice versa. Polyanions play an important role within both systems, with several components interacting with these complex molecules. These molecules include polyphosphates, phospholipids, and GAGs. The net negative charge of GAGs has been shown to modulate contact activation of the intrinsic pathway of coagulation, through activation of FXII,^49^ and it has been observed that FXI can also autoactivate in the presence of polyanionic surfaces such as DXS and sulfatides.^50^ It has been shown that sufficient inhibition of contact pathway proteases by C1‐INH often requires the presence of GAGs, with inhibition of FXIa being enhanced by DXS, heparin, and heparan sulfate.^51^ Complement regulatory mechanisms often occur through interactions with polyanionic molecules^42^; thus, it seems logical to suspect that the presence of negatively charged surfaces may be a junction at which both complement and coagulation interact.

In this preliminary study, we have shown a novel intercommunication between mechanisms of the intrinsic pathway of coagulation and the alternative pathway of complement. We employed the polyanion DXS, a commonly used synthetic GAG, to induce autoactivation of FXI. By titrating physiological concentrations of FP, we showed that cleavage of the chromogenic substrate by FXIa is reduced in a dose‐dependent manner, and this reached almost maximum effect at the highest concentration (25 µg/mL) of FP. To further understand these interactions, DXS, FXI, and FP were incubated and subjected to SDS‐PAGE to determine if FP was acting as a substrate or as a modulator of FXI autoactivation. We determined that autoactivation of FXI was able to occur in the presence of FP and the generated FXIa cleaved FP in the presence of DXS, determined by the identification of the cleavage products using LC‐MS. To our knowledge, this is the first‐ever report that FP is a substrate for FXIa. It was also determined that although there was potential for FXIa to cleave FP, it was still able to cleave its natural substrate FIX into FIXa, suggesting that the interaction between FXIa and FP may be a mechanism involved in inflammatory pathways.

We next explored the effect of FP on FXIa amidolytic activity using Michaelis‐Menten kinetics. FXIa activity was not affected by the presence of FP alone; however, FXIa activity was modulated by DXS, indicated by decreased V_max_, *k*_cat_, and *K*_m_. This modulatory effect of DXS was partially reversed by FP, demonstrated by the rescue of the *k*_cat_, and the increase in *K*_m_ and V_max_ caused by the presence of FP. These data support the substrate specificity change of FXIa and suggest that FP can interact with FXIa in a surface‐dependent manner.

We determined using SPR that there is a direct interaction between FXIa and FP, as both FXI and FXIa bind to FP with high affinity, with K_D_ values in the nanometer and picometer ranges, respectively. Although this binding is determined in a purified system and many other important components are not present, the high affinity does suggest these interactions are physiological and likely to play a functional role in FP and/or FXIa activity. There is evidence to suggest that the interaction between FXI and HK is not permanent and that FXI and PK compete for HK binding, with overlapping binding sites,^52^, ^53^ which may reveal an opportunistic mode of action for FP and FXIa to interact.

To test this further in a purified assay that more closely represents a physiological system, an assay was optimized to determine the effects on FXa generation downstream of FXIa activation of FIX in the presence of phospholipid vesicles. In this purified system, FP appears to modulate FXa generation downstream of FXIa activation of FX in the presence and absence of phospholipid vesicles. As this study has already observed that FIX cleavage by FXIa is still possible in the presence of DXS and FP, it may be that FP is interfering with FIXa cleavage of FX.

It has been shown that FXI deficiency improves survival in a murine model of sepsis caused by cecal ligation and puncture^54^, ^55^ and that FXI deficiency alters the cytokine response.^56^ However, it has recently been demonstrated that FXI appears to play a role in host defense during murine sepsis,^57^ with FXI knockout mice exhibiting enhanced inflammatory responses and lower survival rates when compared to wild‐type mice during *Streptococcus pneumonia* challenge. These studies highlight the important differences in the use of experimental models, and that caution in the interpretation of data needs to be applied. Silasi et al^58^ have also shown that inhibition of FXI in a baboon model of bacterial sepsis is protective, resulting in decreased markers of inflammation, including those related to complement activation, supporting the theory that crosstalk between complement and coagulation may occur at the axis of FXI.

The effect of this novel interaction on the thrombin feedback loop, as described by Gailani and Broze,^32^ is unknown. They stated that GAGs are required for efficient activation of FXI by thrombin. This supports our findings that GAGs play a role in substrate specificity in the coagulation cascade. Gailani and Broze also mention that in the presence of HK, FXI activation appears to be reduced by 40% to 50%; however, this in itself may be indicative of substrate specificity of FXIa being redirected to HK. It has previously been demonstrated that FXIa can cleave HK,^59^ again supporting the findings in this study.

Novel interactions of FXIa have recently emerged, reiterating the fact that crosstalk mechanisms are still not defined. FXIa can cleave prochemerin in contact‐activated plasma to ultimately generate a potent chemoattractant, chemerin,^33^ providing a new link between coagulation and inflammation. FXI has also recently been shown to be implicated in complement, by neutralizing the complement factor H, a major inhibitor of the alternative pathway of complement y.^35^ These findings along with our data support the hypothesis that FXIa may have alternative substrates, and that FXI can independently influence inflammatory pathways. These data show that the coagulation cascade and the mechanisms of interaction between coagulation and inflammation are not yet fully defined, reiterating the need for further work to understand how they may implicate one another. This is especially important, as novel anticoagulant therapeutics are currently aimed at inhibiting FXIa, yet the implications that these new therapies may have on inflammatory mechanisms are not fully understood.

More physiological surfaces need to be explored to determine if the same effect can be exhibited on different surfaces. There are still many avenues to explore, as it is not known whether patients with FP deficiency display bleeding or thrombotic phenotypes. It is clear that the mechanisms behind FXI interactions during sepsis and induced coagulopathies are not fully understood, but there appears to be an important link with the alternative complement pathway that needs further investigation.

**FIGURE 5:**
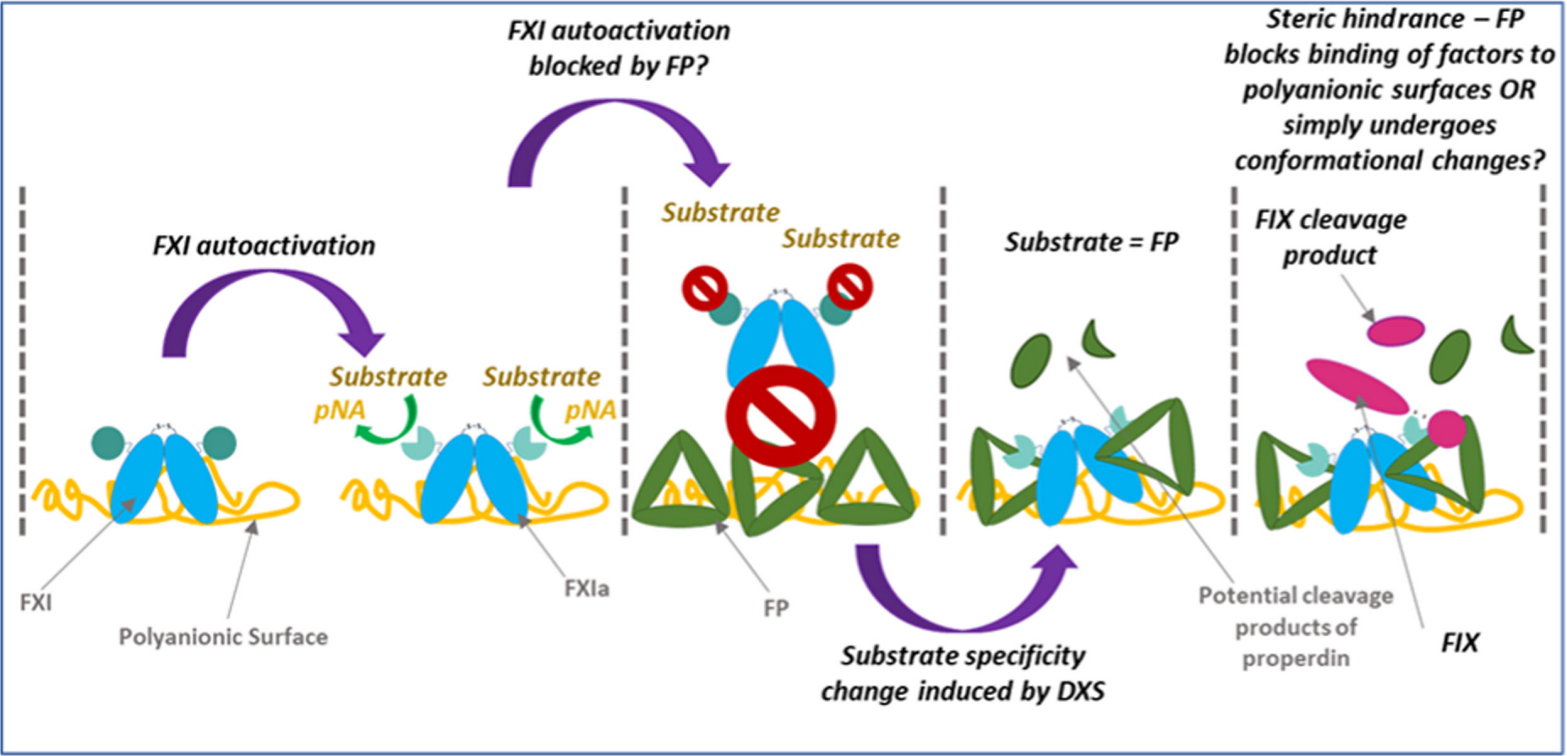
The mystery of the FXIa:FP interaction. FXI autoactivates in the presence of DXS, resulting in the cleavage of a chromogenic substrate and to the release of pNA. We have theorized that FP may block the cleavage of the chromogenic substrate due to a substrate specificity change of FXIa, which leads to the cleavage of FP. However, it may also be due to conformational changes in FP upon surface interactions leading to exposure of cryptic cleavage sites. Abbreviations: DXS, dextran sulfate; FP, factor P; FXI, factor XI; FXIa, activated factor XI; pNA, p‐nitroaniline

It is most likely that FP, a highly positively charged protein, is interacting directly with DXS, coating the anionic surface. FP may undergo conformational changes, making it more susceptible to cleavage by FXIa. We demonstrated an interaction between FP and the mechanism of FXa generation via the intrinsic pathway, which was not surface dependent, however this was more obvious when PL were present in the reactions; thus, steric hindrance may be playing a role in the interaction of FP with intrinsic pathway activity, though it is not as simple as this (Figure 5).

There are limitations to this study, including the availability of FP‐deficient plasma and access to a FP knockout mouse model; it is therefore difficult, at this time, to determine whether these interactions are physiological. These observations lead to a range of important questions that need to be answered. This is the first report, to our knowledge, that FP can be cleaved, and therefore the physiological relevance of this mechanism is unknown. If cleavage products are produced, there is potential for implications in inflammatory diseases.

This study demonstrates a novel interaction between the intrinsic coagulation pathway and the alternative pathway of complement. FXIa is a current target for novel anticoagulation therapeutics, and this study shows the potential for off‐target effects of FXI inhibitors in the treatment of thrombotic disorders.

## RELATIONSHIP DISCLOSURE

The authors declare no competing financial interest relating to this work.

## AUTHOR CONTRIBUTIONS

SLH and HP were responsible for the study design. SLH performed experiments, analyzed data, and prepared the manuscript. LH designed and optimized conditions for experiments, interpreted data, and edited the manuscript. CW, MA, RA, RF, and HP interpreted data and edited the manuscript.

## Supporting information

Supplementary Material

## References

[1] Krem MM , Di Cera E . Evolution of enzyme cascades from embryonic development to blood coagulation. Trends Biochem Sci. 2002;27(2):67‐74. 11852243 10.1016/s0968-0004(01)02007-2

[2] de Bont CM , Boelens WC , Pruijn GJM . NETosis, complement, and coagulation: a triangular relationship. Cell Mol Immunol. 2019;16(1):19‐27. 29572545 10.1038/s41423-018-0024-0 PMC6318284

[3] Elvington M , Liszewski MK , Atkinson JP . Evolution of the complement system: from defense of the single cell to guardian of the intravascular space. Immunol Rev. 2016;274(1):9‐15. 27782327 10.1111/imr.12474 PMC5108576

[4] Diebolder CA , Beurskens FJ , de Jong RN , et al. Complement is activated by IgG hexamers assembled at the cell surface. Science. 2014;343(6176):1260‐1263. 24626930 10.1126/science.1248943 PMC4250092

[5] Kjaer TR , Thiel S , Andersen GR . Toward a structure‐based comprehension of the lectin pathway of complement. Mol Immunol. 2013;56(3):222‐231. 23810291 10.1016/j.molimm.2013.05.220

[6] Turner NA , Moake J . Assembly and activation of alternative complement components on endothelial cell‐anchored ultra‐large von Willebrand factor links complement and hemostasis‐thrombosis. PLoS One. 2013;8(3):e59372. 23555663 10.1371/journal.pone.0059372 PMC3612042

[7] Fearon DT , Austen KF . Properdin: binding to C3b and stabilization of C3b‐dependent C3 convertase. J Exp Med. 1975;142(4):856‐863. 1185108 10.1084/jem.142.4.856 PMC2189935

[8] Bettoni S , Bresin E , Remuzzi G , Noris M , Donadelli R . Insights into the effects of complement factor H on the assembly and decay of the alternative pathway C3 proconvertase and C3 convertase. J Biol Chem. 2016;291(15):8214‐8230. 26903516 10.1074/jbc.M115.693119 PMC4825022

[9] Pangburn MK . Analysis of the natural polymeric forms of human properdin and their functions in complement activation. J Immunol. 1989;142(1):202‐207. 2909614

[10] Cortes C , Ohtola JA , Saggu G , Ferreira VP . Local release of properdin in the cellular microenvironment: role in pattern recognition and amplification of the alternative pathway of complement. Front Immunol. 2013;3:412. 23335922 10.3389/fimmu.2012.00412 PMC3547370

[11] Whaley K . Biosynthesis of the complement components and the regulatory proteins of the alternative complement pathway by human peripheral blood monocytes. J Exp Med. 1980;151(3):501‐516. 6444659 10.1084/jem.151.3.501 PMC2185797

[12] Reis ES , Barbuto JA , Isaac L . Human monocyte‐derived dendritic cells are a source of several complement proteins. Inflamm Res. 2006;55(5):179‐184. 16830104 10.1007/s00011-006-0068-y

[13] Li K , Fazekasova H , Wang N , et al. Expression of complement components, receptors and regulators by human dendritic cells. Mol Immunol. 2011;48(9–10):1121‐1127. 21397947 10.1016/j.molimm.2011.02.003 PMC3084445

[14] Stover CM , Luckett JC , Echtenacher B , et al. Properdin plays a protective role in polymicrobial septic peritonitis. J Immunol. 2008;180(5):3313‐3318. 18292556 10.4049/jimmunol.180.5.3313

[15] Peake PW , O'Grady S , Pussell BA , Charlesworth JA . Detection and quantification of the control proteins of the alternative pathway of complement in 3T3‐L1 adipocytes. Eur J Clin Invest. 1997;27(11):922‐927. 9395788 10.1046/j.1365-2362.1997.2090759.x

[16] Pattrick M , Luckett J , Yue L , Stover C . Dual role of complement in adipose tissue. Mol Immunol. 2009;46(5):755‐760. 18954909 10.1016/j.molimm.2008.09.013

[17] Wirthmueller U , Dewald B , Thelen M , et al. Properdin, a positive regulator of complement activation, is released from secondary granules of stimulated peripheral blood neutrophils. J Immunol. 1997;158(9):4444‐4451. 9127010

[18] Camous L , Roumenina L , Bigot S , et al. Complement alternative pathway acts as a positive feedback amplification of neutrophil activation. Blood. 2011;117(4):1340‐1349. 21063021 10.1182/blood-2010-05-283564

[19] Sjoholm AG . Inherited complement deficiency states: implications for immunity and immunological disease. APMIS. 1990;98(10):861‐874. 2147105 10.1111/j.1699-0463.1990.tb05008.x

[20] Ivanov I , Verhamme IM , Sun M‐F , et al. Protease activity in single‐chain prekallikrein. Blood. 2020;135(8):558‐567. 31800958 10.1182/blood.2019002224 PMC7033373

[21] Colman RW , Schmaier AH . Contact system: a vascular biology modulator with anticoagulant, profibrinolytic, antiadhesive, and proinflammatory attributes. Blood. 1997;90(10):3819‐3843. 9354649

[22] Puy C , Tucker EI , Wong ZC , et al. Factor XII promotes blood coagulation independent of factor XI in the presence of long‐chain polyphosphates. J Thromb Haemost. 2013;11(7):1341‐1352. 23659638 10.1111/jth.12295 PMC3714337

[23] Visser M , van Oerle R , ten Cate H , et al. Plasma kallikrein contributes to coagulation in the absence of factor XI by activating factor IX. Arterioscler Thromb Vasc Biol. 2020;40(1):103‐111. 31766871 10.1161/ATVBAHA.119.313503

[24] Noubouossie D , Henderson MW , Mooberry MJ , et al. Red blood cell microvesicles activate the contact system leading to factor IX activation via two independent pathways. Blood. 2020;135(10):755‐765. 31971571 10.1182/blood.2019001643 PMC7059516

[25] Kearney KJ , Butler J , Posada OM , et al. Kallikrein directly interacts with and activates factor IX, resulting in thrombin generation and fibrin formation independent of factor XI. Proc Natl Acad Sci U S A. 2021;118(3):e2014810118. 33397811 10.1073/pnas.2014810118 PMC7826336

[26] Gailani D , Broze GJ . Factor XII‐independent activation of factor XI in plasma: effects of sulfatides on tissue factor‐induced coagulation. Blood. 1993;82(3):813‐819. 8338946

[27] Mohammed BM , Matafonov A , Ivanov I , et al. An update on factor XI structure and function. Thromb Res. 2018;161:94‐105. 29223926 10.1016/j.thromres.2017.10.008 PMC5776729

[28] Renne T , Gailani D , Meijers JCM , Muller‐Esterl W . Characterization of the H‐kininogen‐binding site on factor XI ‐ a comparison of factor XI and plasma prekallikrein. J Biol Chem. 2002;277(7):4892‐4899. 11733491 10.1074/jbc.M105221200

[29] Macfarlane RG . Enzyme cascade in blood clotting mechanism + its function as biochemical amplifier. Nature. 1964;202(493):498‐499. 14167839 10.1038/202498a0

[30] Davie EW , Ratnoff OD . Waterfall sequence for intrinsic blood clotting. Science. 1964;145(363):1310‐2000. 14173416 10.1126/science.145.3638.1310

[31] Wu WM , Sinha D , Shikov S , et al. Factor XI homodimer structure is essential for normal proteolytic activation by factor XIIa, thrombin, and factor XIa. J Biol Chem. 2008;283(27):18655‐18664. 18441012 10.1074/jbc.M802275200 PMC2441546

[32] Gailani D , Broze GJ . Factor XI activation in a revised model of blood coagulation. Science. 1991;253(5022):909‐912. 1652157 10.1126/science.1652157

[33] Ge XM , Yamaguchi Y , Zhao L , et al. Prochemerin cleavage by factor XIa links coagulation and inflammation. Blood. 2018;131(3):353‐364. 29158361 10.1182/blood-2017-07-792580 PMC5774209

[34] Matafonov A , Cheng Q , Geng Y , et al. Evidence for factor IX‐independent roles for factor XIa in blood coagulation. J Thromb Haemost. 2013;11(12):2118‐2127. 24152424 10.1111/jth.12435 PMC3947433

[35] Puy C , Pang J , Reitsma SE , et al. Cross‐talk between the complement pathway and the contact activation system of coagulation: activated factor XI neutralizes complement factor H. J Immunol. 2021;206(8):1784‐1792. 33811105 10.4049/jimmunol.2000398 PMC8030746

[36] Ghebrehiwet B , Silverberg M , Kaplan AP . Activation of the classical pathway of complement by Hageman‐factor fragment. J Exp Med. 1981;153(3):665‐676. 7252410 10.1084/jem.153.3.665 PMC2186101

[37] Irmscher S , Doring N , Halder LD , et al. Kallikrein cleaves C3 and activates complement. J Innate Immun. 2018;10(2):94‐105. 29237166 10.1159/000484257 PMC6757171

[38] Dobo J , Major B , Kekesi KA , et al. Cleavage of kininogen and subsequent bradykinin release by the complement component: mannose‐binding lectin‐associated serine protease (MASP)‐1. PLoS One. 2011;6(5):e20036. 21625439 10.1371/journal.pone.0020036 PMC3100311

[39] Davis AE , Lu FX , Mejia P . C1 inhibitor, a multi‐functional serine protease inhibitor. Thromb Haemost. 2010;104(5):886‐893. 20806108 10.1160/TH10-01-0073

[40] Davis AE , Mejia P , Lu FX . Biological activities of C1 inhibitor. Mol Immunol. 2008;45(16):4057‐4063. 18674818 10.1016/j.molimm.2008.06.028 PMC2626406

[41] Meri S , Pangburn MK . Discrimination between activators and nonactivators of the alternative pathway of complement: regulation via a sialic acid/polyanion binding site on factor H. Proc Natl Acad Sci U S A. 1990;87(10):3982‐3986. 1692629 10.1073/pnas.87.10.3982 PMC54028

[42] Wijeyewickrema LC , Lameignere E , Hor L , et al. Polyphosphate is a novel cofactor for regulation of complement by a serpin, C1 inhibitor. Blood. 2016;128(13):1766‐1776. 27338096 10.1182/blood-2016-02-699561 PMC5043130

[43] Yang AZ , Chen FW , He C , et al. The Procoagulant activity of apoptotic cells is mediated by interaction with factor XII. Front Immunol. 2017;8:1188. 28993777 10.3389/fimmu.2017.01188 PMC5622377

[44] Mold C . Effect of membrane phospholipids on activation of the alternative complement pathway. J Immunol. 1989;143(5):1663‐1668. 2527270

[45] Byrnes JR , Wilson C , Boutelle AM , et al. The interaction between fibrinogen and zymogen FXIII‐A2B2 is mediated by fibrinogen residues γ390‐396 and the FXIII‐B subunits. Blood. 2016;128(15):1969‐1978. 27561317 10.1182/blood-2016-04-712323 PMC5064719

[46] Ahnström J , Andersson HM , Canis K , et al. Activated protein C cofactor function of protein S: a novel role for a γ‐carboxyglutamic acid residue. Blood. 2011;117(24):6685‐6693. 21508412 10.1182/blood-2010-11-317099

[47] Jaques LB . Protamine–antagonist to heparin. Can Med Assoc J. 1973;108(10):1291‐1297. 4122234 PMC1941450

[48] Wadsworth A , Maltaner F , Maltaner E . The inhibition of complementary activity by anticoagulants. J Immunol. 1937;33(4):297‐303.

[49] Samuel M , Pixley RA , Villanueva MA , Colman RW , Villanueva GB . Human factor XII (Hageman factor) autoactivation by dextran sulfate. Circular dichroism, fluorescence, and ultraviolet difference spectroscopic studies. J Biol Chem. 1992;267(27):19691‐19697. 1527088

[50] Naito K , Fujikawa K . Activation of human blood‐coagulation factor‐XI independent of factor‐XII ‐ factor‐XI is activated by thrombin and factor‐XIa in the presence of negatively charged surfaces. J Biol Chem. 1991;266(12):7353‐7358. 2019570

[51] Wuillemin WA , Eldering E , Citarella F , deRuig CP , tenCate H , Hack CE . Modulation of contact system proteases by glycosaminoglycans ‐ selective enhancement of the inhibition of factor XIa. J Biol Chem. 1996;271(22):12913‐12918. 8662679 10.1074/jbc.271.22.12913

[52] Thompson RE , Mandle R , Kaplan AP . Studies of binding of prekallikrein and factor XI to high molecular weight kininogen and its light chain. Proc Natl Acad Sci U S A. 1979;76(10):4862‐4866. 291905 10.1073/pnas.76.10.4862 PMC413037

[53] Renné T , Dedio J , Meijers JC , Chung D , Müller‐Esterl W . Mapping of the discontinuous H‐kininogen binding site of plasma prekallikrein. Evidence for a critical role of apple domain‐2. J Biol Chem. 1999;274(36):25777‐25784. 10464316 10.1074/jbc.274.36.25777

[54] Tucker EI , Gailani D , Hurst S , Cheng QF , Hanson SR , Gruber A . Survival advantage of coagulation factor XI‐deficient mice during peritoneal sepsis. J Infect Dis. 2008;198(2):271‐274. 18491973 10.1086/589514 PMC2654284

[55] Tucker EI , Verbout NG , Leung PY , et al. Inhibition of factor XI activation attenuates inflammation and coagulopathy while improving the survival of mouse polymicrobial sepsis. Blood. 2012;119(20):4762‐4768. 22442348 10.1182/blood-2011-10-386185 PMC3367876

[56] Bane CE , Ivanov I , Matafonov A , et al. Factor XI deficiency alters the cytokine response and activation of contact proteases during polymicrobial sepsis in mice. PLoS One. 2016;11(4):e0152968. 27046148 10.1371/journal.pone.0152968 PMC4821616

[57] Stroo I , Zeerleder S , Ding C , et al. Coagulation factor XI improves host defence during murine pneumonia‐derived sepsis independent of factor XII activation. Thromb Haemost. 2017;117(8):1601‐1614. 28492700 10.1160/TH16-12-0920

[58] Silasi R , Keshari RS , Lupu C , et al. Inhibition of contact‐mediated activation of factor XI protects baboons against S aureus‐induced organ damage and death. Blood Adv. 2019;3(4):658‐669. 30808684 10.1182/bloodadvances.2018029983 PMC6391670

[59] Mauron T , Lämmle B , Wuillemin WA . High molecular weight kininogen is cleaved by FXIa at three sites: Arg409‐Arg410, Lys502‐Thr503 and Lys325‐Lys326. Thromb Haemost. 2000;83(5):709‐714. 10823267

