# Supplementary material for: Novel interaction of properdin and coagulation factor XI: Crosstalk between complement and coagulation
Source: Res Pract Thromb Haemost. 2022 May 24;6(4):e12715. doi: 10.1002/rth2.12715 (PMC9130567; doi:10.1002/rth2.12715)

**Supporting Information**

**Methods**

**Liquid chromatography-mass spectrometry digest protocol**

Gel bands were excised and chopped into small pieces (~ 1 mm^3^), covered with 30% ethanol in a 1.5 mL microcentrifuge and heated to 56°C for 30 minutes with shaking. The supernatant was removed and replaced with fresh ethanol solution was again heated to 56°C for 30 minutes with shaking. This was repeated until all Coomassie stain was removed from the gel. The gel slices were then dehydrated by covering with 100% acetonitrile and left for 5 minutes before the supernatant was discarded and replaced with a fresh aliquot of acetonitrile. Proteins were reduced by adding 100 µL 20 mM DTT solution before incubation at 57°C for 1 hour with shaking.

The supernatant was removed and once the gel pieces were at room temperature proteins were alkylated by adding 100 µL 55 mM iodoacetic acid. The samples were then incubated at room temperature in the dark for 30 minutes with shaking. After removing the supernatant, the gel slices were then covered with 100% acetonitrile and left for 5 minutes. The acetonitrile was removed, and the gel pieces were left to dry in a laminar flow hood for 60 minutes. Once dry, the gel slices were cooled on ice then they were then covered with ice-cold trypsin solution (20 ng/µL in 25 mM ammonium bicarbonate) and left on ice for 20 minutes to rehydrate. Excess trypsin solution was removed, and the gel slices were covered with a minimal amount of 25 mM ammonium bicarbonate. After briefly vortexing and centrifuging, the gel slices were incubated at 37°C with shaking for 18 hours. The resulting digest was vortexed and centrifuged. The supernatant was recovered and added to an Eppendorf containing 5 µL acetonitrile/ water/ formic acid (60/35/5; v/v).  50 µL acetonitrile/ water/ formic acid (60/35/5; v/v) was added to the gel slices and vortexed for an additional 10 minutes. The supernatant was pooled with the previous wash and one additional wash of the gel slices was performed. The pool of three washes was dried by vacuum centrifugation. The peptides were reconstituted in 20 µL 0.1% aqueous trifluoroacetic acid prior to analysis.

*Liquid chromatography-mass spectrometry analysis*

An aliquot of 10 µL was used for LC-MS. LC separation of the peptide mixtures was performed on an ACQUITY M-Class UPLC (Waters UK, Manchester). 1 µL of each sample was loaded onto a Symmetry C18 trap column (180 µM i.d. * 20 mm) and washed with 1% acetonitrile/0.1% formic acid for 5 min at 5 µL/min.  After valve switching, the peptides were then separated on a HSS T3 C18, 75 µm i.d. x 150 mm analytical column (Waters UK, Manchester) by gradient elution of 1-60% solvent B in A over 30 minutes. at 0.3 µL/min. Solvent A was 0.1% formic acid in water, solvent B was 0.1% formic acid in acetonitrile.

The column eluant was directly interfaced to a quadrupole-orthogonal time of flight mass spectrometer (Xevo G2-XS Q-TOF, Waters UK, Manchester) via a Z-spray nanoflow electrospray source.  The MS was operated in positive TOF mode using a capillary voltage of 3.0 kV, cone voltage of 40 V, source offset of 80 V, backing pressure of 3.58 mbar. The source temperature was 80°C.  Argon was used as the buffer gas at a pressure of 8.6 × 10^-3^ mbar in the trap and transfer regions.    Mass calibration was performed using [Glu]-fibrinopeptide (GFP) at a concentration of 250 fmol/µL.  GFP was also used as a lock mass calibrant with a one second lock spray scan taken every 30 seconds during acquisition. Ten scans were averaged to determine the lock mass correction factor.  Data acquisition was using data dependent analysis with a 0.2 s scan MS over *m/z*350-2000 being followed by five 0.5 s MS/MS taken of the five most intense ions in the MS spectrum.  CE applied was dependent upon charge state and mass of the ion selected.  Dynamic exclusion of 60 s was used. Data processing was performed using the MassLynx v4.1 suite of software supplied with the mass spectrometer. Peptide MS/MS data were processed with PEAKS Studio (Bioinformatic Solutions Inc, Waterloo, Ontario, Canada) and searched against a database of human proteins downloaded from Uniprot (2018_11). The false discovery rate was set to 1%.

LC-MS results revealed that the cleavage products are FP.

**SPR**

*Protocol*

Assay running buffer (RB) contained 10 mM 4-(2-hydroxyethyl)-1-piperazineethanesulfonic acid (HEPES), 150 mM NaCl, 2 mM MgCl_2_, 1.5 mM CaCl_2_, 0.04 mM ZnCl_2_ and 0.05% Tween®20 pH 7.4 and was filter-sterilised and degassed. To reduce refractive index (RI) spikes, FXI and FXIa analytes were dialysed into RB. SPR was performed at a temperature of 22°C. A COOHV chip was installed according to manufacturer’s instructions and primed three times with RB. The theoretical R_MAX_ was used to calculate the level of immobilisation of FP.

Buffer exchange was performed at 4°C with stirring, buffer was changed after for 4 hours and samples were dialysed for a further 16 hours. Analyte concentration was determined using a Nanodrop1000 (Thermo Fisher, Waltham, MA).

The surface was preconditioned for amine coupling using two bursts of 6 µL 10 mM HCl, 50 mM NaOH and 0.1% SDS at 100 µL/min followed by normalising with 100% DMSO and priming three times.

**Results**

**Protamine Sulfate Reduces Cleavage of S-2288 by FXI in the presence of DXS.**


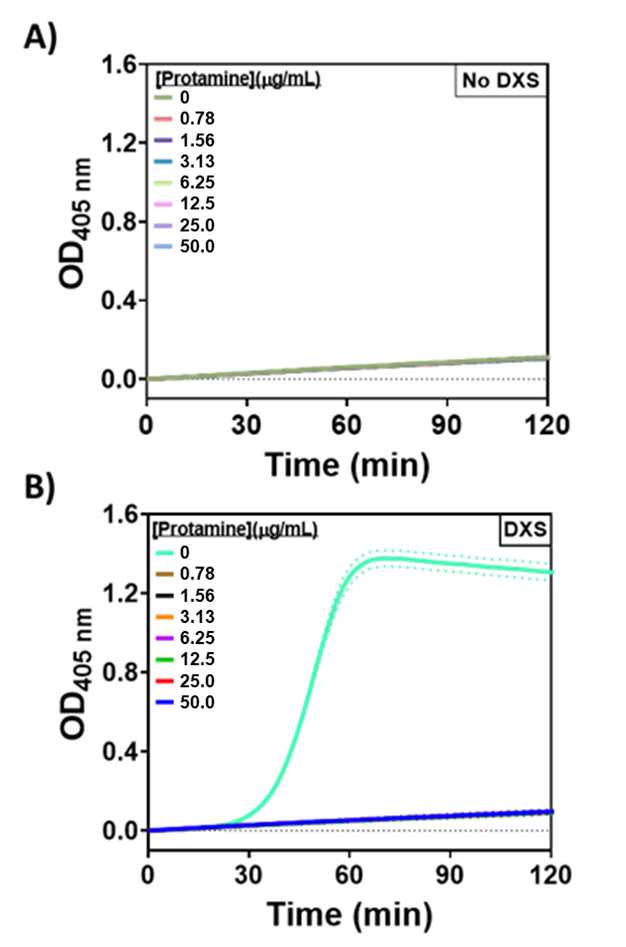


**Supporting Information Figure 1. Protamine sulfate inhibits FXI autoactivation by DXS.** A chromogenic assay was employed to determine the autoactivation of 30 nM FXI in the absence (A) and presence (B) of 0.6 µg/mL DXS_500kDa_ and the effect of a titration (0.78 - 50 µg/mL) of protamine sulfate on this process. FXI autoactivation was measured by cleavage of 500 µM chromogenic substrate S-2288 over 120 minutes. Optical density was read at 405 nm, at 12 second intervals. Data are expressed as mean ± SEM constructed of one experiment run in triplicate. Error bars may not be visible due to small values.

**LC-MS reveals FP cleavage bands.**

**Supporting Information Table 1. Results of LC-MS analysis of SDS-PAGE.**


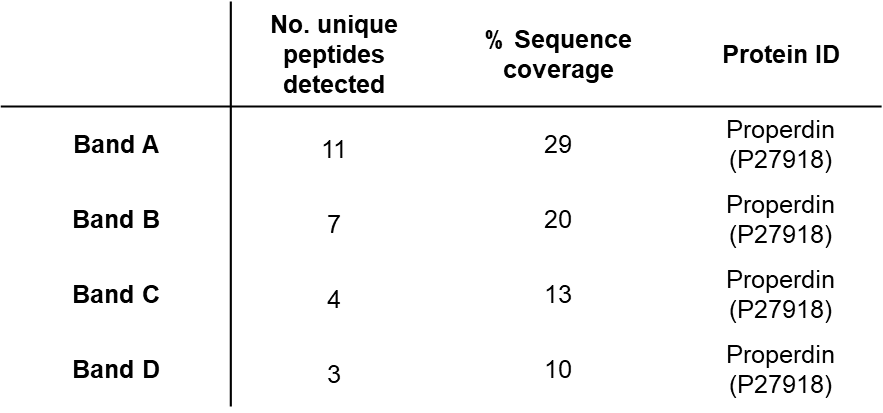

Supplement: Supplementary file 1 — Supplementary Material [file RTH2-6-e12715-s001.docx]
